# Prognostic value of baseline plasma D-dimer levels in sepsis: a prospective cohort study

**DOI:** 10.1016/j.plabm.2025.e00498

**Published:** 2025-08-23

**Authors:** Xiaoxiao Qu, Shishi Wang, Xuanmei Ye, Guosong Jiang, Mihereguli Kuerban, Qipeng Xie

**Affiliations:** a Department of Clinical Laboratory, The Second Affiliated Hospital & Yuying Children's Hospital of Wenzhou Medical University, Wenzhou, 325000, Zhejiang, China; b Department of Pulmonary and Critical Care Medicine, The 1st People's Hospital of Zhaotong City&The Zhaotong Affiliated Hospital of Kunming Medical University, Zhaotong, 657000, Yunnan, China; c School of Medical Technology, XinJiang HeTian College, Hetian, Xinjiang, 848000, China

**Keywords:** Sepsis, D-dimer, All-cause mortality, Septic shock, Prognostic biomarker, Intensive care unit, Prospective cohort study

## Abstract

**Background:**

Plasma D-dimer, a fibrin degradation product, reflects coagulation activation and is often elevated in critically ill patients. Its prognostic significance in sepsis, particularly for short-term outcomes, remains unclear.

**Methods:**

In this prospective cohort study, we enrolled 175 adult ICU patients with sepsis (Sepsis-3 criteria) from March 2024 to February 2025. Plasma D-dimer levels were measured at ICU admission and daily for five days. D-dimer levels were categorized into quartiles. The primary outcome was 30-day all-cause mortality; secondary outcome was in-hospital septic shock. Associations were analyzed using Cox regression, Kaplan–Meier analysis, and subgroup analysis.

**Results:**

Elevated admission D-dimer levels were significantly associated with increased risks of 30-day mortality and septic shock. Each 1 μg/mL increase in D-dimer was linked to a 6 % higher mortality risk (HR = 1.06; 95 % CI: 1.02–1.11; P = 0.008) and an 8 % higher septic shock risk (HR = 1.08; 95 % CI: 1.03–1.12; P < 0.001), after adjusting for confounders. Patients in the highest quartile had the worst outcomes. A significant interaction with serum amyloid A (SAA) was observed for mortality (P = 0.043), but not for septic shock.

**Conclusion:**

Baseline plasma D-dimer levels independently predict 30-day mortality and septic shock in sepsis. D-dimer may serve as a valuable early biomarker for risk stratification in sepsis management.

## 1 Introduction

Sepsis, defined as life-threatening organ dysfunction resulting from a dysregulated host response to infection, remains a major global health burden [1]. Recent estimates indicate that sepsis affects approximately 49 million individuals annually and contributes to 11 million deaths worldwide, accounting for nearly 20 % of global mortality [2]. Despite advances in critical care, sepsis continues to be associated with high morbidity and mortality, particularly among patients admitted to intensive care units (ICU) [3,4]. Early identification of high-risk individuals is essential for optimizing clinical decision-making and improving outcomes. However, reliable, accessible biomarkers for early prognostic evaluation remain limited.

Coagulation abnormalities are central to the pathophysiology of sepsis and contribute to complications such as microvascular thrombosis, disseminated intravascular coagulation (DIC), and multi-organ failure [5]. D-dimer, a fibrin degradation product, serves as a sensitive indicator of coagulation activation and fibrinolysis [6]. Elevated D-dimer levels have been documented in various critical conditions, including venous thromboembolism, cardiovascular diseases, and severe infections [[7], [8], [9]]. Recent studies have suggested that D-dimer may be associated with increased disease severity and adverse outcomes in patients with sepsis [[10], [11], [12]]. However, most existing studies are retrospective in design and suffer from substantial heterogeneity in sample populations, limited endpoint definitions, and inadequate control of confounding variables. In particular, there is a lack of systematic evaluation of composite outcomes, such as the combination of mortality and septic shock. Moreover, the inherent limitations of retrospective studies restrict their ability to establish causal inferences, making it difficult to determine the clinical utility of D-dimer as an independent prognostic marker.

This prospective cohort study enrolled patients with sepsis admitted to the intensive care unit (ICU) to investigate the independent association between plasma D-dimer levels at ICU admission and 30-day all-cause mortality and septic shock. Unlike most previous retrospective studies that focused on a single clinical endpoint, our study separately examined both 30-day mortality and septic shock, with D-dimer levels clearly defined at ICU admission—thereby highlighting its potential as an early prognostic marker in sepsis. To further assess the clinical significance of temporal changes in D-dimer levels, serial measurements were performed daily for five consecutive days following ICU admission. Given its simplicity, low cost, and widespread use in routine clinical practice, D-dimer testing represents a feasible tool for early identification of high-risk patients and may help guide subsequent management decisions. Multivariable regression models were used to rigorously control for potential confounders, thereby enhancing the robustness and clinical relevance of the findings. Inflammation and coagulation are closely intertwined in the pathophysiology of sepsis. To account for this interplay, we included serum amyloid A (SAA) and soluble suppression of tumorigenicity 2 (sST2) as representative inflammatory markers. These biomarkers have been previously associated with prognosis in critical illness. In this study, they were incorporated primarily as covariates in multivariable models and for subgroup analyses, to explore whether the prognostic value of D-dimer was independent of or modified by systemic inflammation. This study is expected to provide foundational evidence supporting the use of D-dimer in early risk stratification among patients with sepsis and offer insights for future mechanistic and interventional research.

## 2 Methods

### 2.1 Study design and participants

This was a prospective cohort study with supplemental retrospective data collection, conducted at the Second Affiliated Hospital & Yuying Children's Hospital of Wenzhou Medical University. All patients admitted to the intensive care unit (ICU) between March 1, 2024, and February 28, 2025, were screened for eligibility. Inclusion criteria were as follows: (1) age ≥18 years; (2) diagnosis of sepsis based on the Sepsis-3 criteria with a Sequential Organ Failure Assessment (SOFA) score ≥2; and (3) ICU admission [13]. Exclusion criteria included: (1) pre-existing severe hepatic or renal dysfunction; (2) known allergies to antibiotics or other medications; and (3) active bleeding tendency or current use of anticoagulant therapy [14]. Patients were stratified based on clinical outcomes. Those who died within 30 days or developed septic shock during hospitalization were categorized into the Adverse Event Group, while patients who survived without developing septic shock comprised the Non-Adverse Event Group. The primary outcome was 30-day all-cause mortality; the secondary outcome was the incidence of in-hospital septic shock. To evaluate the prognostic value of baseline D-dimer levels, we conducted daily measurements from ICU admission through Day 5 to observe potential trends over time. These serial values were used to analyze temporal trends and their associations with clinical outcomes.

**Fig. 1:**
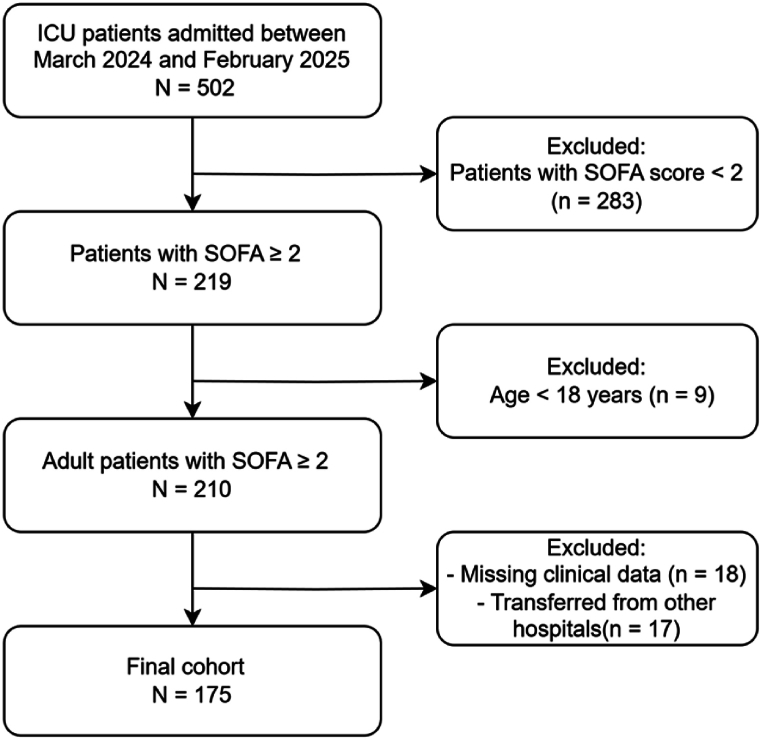
Flowchart of patient enrollment and exclusion criteria.

The patient enrollment process is illustrated in Fig. 1. The study protocol was approved by the Ethics Committee of the Second Affiliated Hospital of Wenzhou Medical University (Approval No. 2023-K-234-01) and was conducted in accordance with the Declaration of Helsinki. Residual serum samples collected during routine care were used for research purposes under ethical approval, with a waiver of informed consent granted due to anonymization and the non-interventional nature of the study.

ICU patients admitted between March 1, 2024, and February 28, 2025, were screened for eligibility. Patients were included if they were adults (≥18 years) and met Sepsis-3 criteria with a SOFA score ≥2. Exclusion criteria included age <18 years, missing clinical data, transfer from other hospitals, pre-existing severe hepatic or renal dysfunction, drug allergies, or anticoagulant use. A total of 175 patients were included in the final cohort.

### 2.2 Data collection and measurements

Demographic characteristics, comorbidities (hypertension, diabetes, Shock), vital signs, laboratory parameters, and SOFA scores were collected from electronic medical records at ICU admission. Venous blood samples were obtained upon admission. Routine laboratory tests were performed immediately, and serum samples were stored at −80 °C for subsequent analyses. Serum soluble suppression of tumorigenicity 2 (sST2) levels were measured using enzyme-linked immunosorbent assay (ELISA) kits (Shanghai Weya Biotechnology Co., Ltd., China). Serum amyloid A (SAA) levels were determined by latex-enhanced immunoturbidimetric assay (Zhejiang Qiangsheng Biotechnology Co., Ltd.) on a Beckman AU5800 analyzer.

### 2.3 D-dimer measurement

Plasma D-dimer levels were measured using the **STA-Liatest D-Di Plus assay** (Diagnostica Stago, France), a latex-enhanced immunoturbidimetric method performed on the **STA-R Evolution analyzer**. Results were reported in **Fibrinogen Equivalent Units (FEU)**, in accordance with the manufacturer's specifications and national standards. The reference interval was 0–0.55 μg/mL, in accordance with the manufacturer's specifications and national standards. Internal quality control (IQC) was performed daily using commercial control materials (Bio-Rad Laboratories, USA). The assay also participated in external quality assessment (EQA) programs organized by the National Center for Clinical Laboratories (NCCL) twice annually.

D-dimer levels were obtained from venous blood samples collected daily for the first 5 consecutive days following ICU admission (Days 1–5). The Day 1 sample was collected at ICU admission before any intervention. All samples were processed under consistent preanalytical conditions.

For statistical analysis, D-dimer levels were evaluated both as continuous variables and as categorical variables stratified by quartiles. Additionally, daily changes in D-dimer levels over the first five days following ICU admission were analyzed to examine their association with clinical outcomes.

### 2.4 Outcome

The primary outcome was 30-day all-cause mortality among patients with sepsis. The secondary outcome was the incidence of septic shock during hospitalization. Patients were prospectively enrolled between March 1, 2024, and February 28, 2025, at our institution. The 30-day observation period was calculated from the date of hospital admission.

Survival status was obtained from the hospital's electronic medical records and, when necessary, supplemented by telephone follow-up for patients who were discharged or had incomplete records. This approach ensured comprehensive and accurate outcome ascertainment.

Septic shock was diagnosed according to the Sepsis-3 definition: sepsis accompanied by persistent hypotension requiring vasopressors to maintain a mean arterial pressure (MAP) ≥65 mmHg and a serum lactate level >2 mmol/L despite adequate fluid resuscitation. Occurrence of septic shock was monitored in real-time and documented by the clinical care team throughout hospitalization.

All clinical data were entered into a prospectively maintained database with standardized data management procedures and routine quality control checks. For patients lost to follow-up, the date of last contact was recorded and included in the survival analysis.

### 2.5 Covariates

Covariates included demographic characteristics, comorbidities, vital signs, and laboratory parameters collected at ICU admission. Categorical variables were sex (male or female), history of hypertension, diabetes mellitus, and presence of septic shock (yes or no). Continuous variables included age, Sequential Organ Failure Assessment (SOFA) score, white blood cell (WBC) count, respiratory rate, serum amyloid A (SAA) level, soluble suppression of tumorigenicity 2 (sST2) level, and plasma D-dimer level. All covariates were selected based on clinical relevance and prior literature and were included in multivariable models to adjust for potential confounding.

### 2.6 Statistical analysis

Patients were categorized into quartiles based on plasma D-dimer levels. Continuous variables were expressed as mean ± standard deviation (SD) or median with interquartile range (IQR), as appropriate based on data distribution. Categorical variables were summarized as counts and percentages (n, %). Group comparisons were performed using one-way analysis of variance (ANOVA) or the Kruskal–Wallis test for continuous variables, and the chi-square test for categorical variables.

Multivariable Cox proportional hazards models were used to estimate hazard ratios (HRs) and 95 % confidence intervals (CIs) for the associations between D-dimer levels and two outcomes: 30-day all-cause mortality and in-hospital septic shock. Three models were constructed: Model 1 was unadjusted; Model 2 was adjusted for age and sex; and Model 3 was additionally adjusted for white blood cell (WBC) count, respiratory rate, hypertension, diabetes mellitus, serum amyloid A (SAA), and soluble suppression of tumorigenicity 2 (sST2). As a sensitivity analysis, serum creatinine was further included in Model 3 to evaluate whether renal function influenced the association between D-dimer and 30-day mortality.

Kaplan–Meier survival curves were generated to compare cumulative 30-day mortality and incidence of septic shock across D-dimer quartiles, with the log-rank test used to assess differences between groups. Subgroup analyses and interaction tests were performed using multivariable Cox models to evaluate potential effect modification by age, sex, SOFA score, SAA levels, and sST2 levels.

The sample size was informed by prior literature reporting 30-day mortality of approximately 30 % in septic ICU patients and estimated hazard ratios for D-dimer ranging from 1.1 to 1.2 [10,15].Assuming α = 0.05 and 80 % power, a minimum of 160–170 patients was considered sufficient to detect a moderate association. The final sample of 175 patients meets this requirement and allows for potential missing data.

All analyses were performed using R software (version 4.2.2) and the Free Statistics Analysis Platform (version 2.1, Beijing, China). A two-sided P value < 0.05 was considered statistically significant.

## 3 Results

### 3.1 Baseline characteristics of patients stratified by D-dimer quartiles

A total of 175 patients with sepsis were included in the analysis, with a mean age of 66.0 ± 15.6 years; 54 patients (30.9 %) were female. The mean SOFA score at ICU admission was 5.9 ± 3.6, and the median serum sST2 level was 3.3 ng/mL (IQR: 2.0–4.8). Overall, 60 patients (34.3 %) developed septic shock during hospitalization.

Baseline characteristics stratified by plasma D-dimer quartiles are summarized in Table 1. Higher D-dimer levels were significantly associated with increased SOFA scores (*P* < 0.001) and elevated respiratory rates (*P* = 0.019). No significant differences were observed across D-dimer quartiles with respect to age, sex, SAA levels, sST2 levels, white blood cell (WBC) count, history of hypertension or diabetes, or the proportion of patients who developed septic shock (*P* > 0.05).

**Table 1:** Baseline characteristics of patients with sepsis according to plasma D-dimer quartiles.

Variables	Total (*N* = 175)	Q1(*N* = 44) <1.26	Q2 (*N* = 43) 1.26–2.18	Q3(*N* = 44) 2.18–5.38	Q4(*N* = 44) >5.38	*P* value
Age.Years	66.0 ± 15.6	66.0 ± 14.6	64.1 ± 14.6	67.0 ± 18.1	66.7 ± 15.0	0.821
Sex, n (%)						0.391
Female	54 (30.9)	10 (22.7)	12 (27.9)	15 (34.1)	17 (38.6)	
Male	121 (69.1)	34 (77.3)	31 (72.1)	29 (65.9)	27 (61.4)	
SAA (mg/L)	1134.9 ± 517.8	1014.4 ± 581.8	1200.4 ± 463.0	1246.0 ± 474.2	1080.4 ± 526.8	0.133
sST2 (ng/ml)	3.3 (2.0, 4.8)	3.0 (1.9, 4.4)	3.4 (2.1, 4.2)	3.3 (1.9, 5.7)	3.6 (2.3, 4.9)	0.796
WBC ( × 109/L)	11.3 (7.7, 17.4)	10.1 (8.7, 12.5)	12.5 (7.5, 17.9)	11.5 (6.9, 17.7)	12.2 (8.2, 17.6)	0.630
SOFA	5.9 ± 3.6	5.0 ± 3.2	5.5 ± 3.0	4.6 ± 3.0	8.4 ± 3.8	<0.001
Respiratory.rate	21.3 ± 9.1	19.1 ± 2.1	22.5 ± 8.6	19.4 ± 4.2	24.3 ± 14.8	0.019
Hypertension, n (%)						0.402
No	93 (53.1)	19 (43.2)	26 (60.5)	25 (56.8)	23 (52.3)	
Yes	82 (46.9)	25 (56.8)	17 (39.5)	19 (43.2)	21 (47.7)	
Diabetes, n (%)						0.835
No	126 (72.0)	30 (68.2)	33 (76.7)	32 (72.7)	31 (70.5)	
Yes	49 (28.0)	14 (31.8)	10 (23.3)	12 (27.3)	13 (29.5)	
SHOCK, n (%)						0.243
No	115 (65.7)	33 (75)	29 (67.4)	29 (65.9)	24 (54.5)	
Yes	60 (34.3)	11 (25)	14 (32.6)	15 (34.1)	20 (45.5)	

Data are presented as mean ± standard deviation (SD), median (interquartile range, IQR), or number (percentage), as appropriate.

Abbreviations: SAA, serum amyloid A; sST2, soluble suppression of tumorigenicity 2; WBC, white blood cell count; SOFA, Sequential Organ Failure Assessment; ICU, intensive care unit.

### 3.2 The association between D-dimer levels and 30-day all-cause mortality

Multivariable Cox regression analysis demonstrated a significant association between plasma D-dimer levels and 30-day all-cause mortality in patients with sepsis (Table 2). In the fully adjusted model (Model 3), each 1 μg/mL increase in D-dimer was associated with a 6 % higher risk of death (HR = 1.06; 95 % CI: 1.02–1.11; P = 0.008). When analyzed by D-dimer quartiles, patients in the highest quartile (Q4, >5.38 μg/mL) had a 132 % higher risk of 30-day mortality compared to those in the lowest quartile (Q1, <1.26 μg/mL) (HR = 2.32; 95 % CI: 1.03–5.23; P = 0.042). In the sensitivity analysis that additionally adjusted for serum creatinine, the association between D-dimer and 30-day mortality remained statistically significant (HR = 1.06, 95 % CI: 1.02–1.11, P = 0.007). In the quartile model, the highest D-dimer group (Q4) continued to show an increased risk of death (HR = 2.34, 95 % CI: 1.02–5.36, P = 0.044), indicating robustness of the association。(Supplementary Table 2). To further examine the non-linear association between D-dimer levels and 30-day mortality, we conducted a restricted cubic spline (RCS) analysis. As shown in Supplementary Fig. 1, the relationship was non-linear (P for non-linearity = 0.045), with the lowest hazard observed at approximately 2.2 μg/mL, followed by a marked increase in mortality risk at higher D-dimer levels.

**Table 2:** Association between plasma D-dimer levels and 30-day all-cause mortality in patients with sepsis.

Categories	Model 1		Model 2		Model 3	
	HR (95 %CI)	*P* value	HR (95 %CI)	*P* value	HR (95 %CI)	*P* value
D-Dimer	1.07 (1.03–1.12)	0.001	1.07 (1.03–1.12)	0.001	1.06 (1.02–1.11)	0.008
D-Dimer Quartile						
Q1 (<1.26)	1(Ref)		1(Ref)		1(Ref)	
Q2 (1.26–2.18)	1.31 (0.54–3.16)	0.547	1.31 (0.54–3.17)	0.544	1.07 (0.43–2.67)	0.888
Q3 (2.18–5.38)	0.54 (0.18–1.62)	0.272	0.51 (0.17–1.52)	0.225	0.34 (0.11–1.09)	0.069
Q4 (>5.38)	2.69 (1.23–5.92)	0.014	2.64 (1.19–5.83)	0.017	2.32 (1.03–5.23)	0.042

Model 1: unadjusted.

Model 2:adjusted for age and sex.

Model 3: adjusted for age, sex, WBC count, respiratory rate, hypertension, diabetes, serum amyloid A (SAA), and soluble suppression of tumorigenicity 2 (sST2).

Abbreviations: HR, hazard ratio; CI, confidence interval; WBC, white blood cell count.

### 3.3 Association between D-dimer levels and risk of septic shock

Higher plasma D-dimer levels were significantly associated with an increased risk of developing septic shock in patients with sepsis (Table 3). In the fully adjusted logistic regression model (Model 3), each 1 μg/mL increase in D-dimer was associated with an 8 % higher odds of septic shock (OR = 1.08; 95 % CI: 1.03–1.12; *P* < 0.001). Compared with patients in the lowest D-dimer quartile (Q1, <1.26 μg/mL), those in the highest quartile (Q4, >5.38 μg/mL) had more than a threefold increased odds of septic shock (OR = 3.08; 95 % CI: 1.46–6.53; *P* = 0.003). A significant linear trend across D-dimer quartiles was observed (*P* for trend = 0.005).

**Table 3:** Association between plasma D-dimer levels and the risk of septic shock in patients with sepsis.

Categories	Model 1		Model 2		Model 3	
	OR (95 %CI)	*P* value	OR (95 %CI)	*P* value	OR (95 %CI)	*P* value
D-Dimer	1.08(1.03–1.12)	<0.001	1.08(1.04–1.12)	<0.001	1.08(1.03–1.12)	<0.001
D-Dimer Quartile						
Q1 (<1.26)	1(Ref)		1(Ref)		1(Ref)	
Q2 (1.26–2.18)	1.54(0.70–3.40)	0.283	1.54(0.70–3.40)	0.283	1.46(0.66–3.23)	0.354
Q3 (2.18–5.38)	1.39(0.64–3.04)	0.403	1.44(0.66–3.16)	0.363	1.42(0.64–3.13)	0.385
Q4 (>5.38)	3.05(1.46–6.37)	0.003	3.13(1.49–6.59)	0.003	3.08(1.46–6.53)	0.003
*P* for trend		0.006		0.005		0.005

Model 1: unadjusted.

Model 2: adjusted for age and sex.

Model 3: adjusted for age, sex, white blood cell (WBC) count, respiratory rate, hypertension, diabetes, serum amyloid A (SAA), and soluble suppression of tumorigenicity 2 (sST2).

Abbreviations: OR, hazard ratio; CI, confidence interval.

### 3.4 Relationship between serial D-dimer measurements and 30-day mortality and septic shock

D-dimer levels on Day 1 were significantly associated with 30-day all-cause mortality. In the fully adjusted model (Model 3), the hazard ratio was 1.12 (95 % CI: 1.02–1.23, *P* = 0.015), indicating that for every 1 μg/mL increase in D-dimer, the risk of death increased by approximately 12 %. In contrast, D-dimer levels from Days 2–5 showed no statistically significant associations with mortality in any of the models (*P* > 0.05) (Table 4). However, No significant associations were observed between D-dimer levels and the occurrence of septic shock at any time point (Day 1–5). In the fully adjusted model, the odds ratios across all time points ranged from 0.96 to 1.06, with all *P* values exceeding 0.05 (Supplementary Table 1).

**Table 4:** Association between daily Plasma D-dimer levels (Day 1–5) and 30-day all-cause mortality in ICU patients with sepsis.

D-dimer Day	N	Model 1		Model 2		Model 3	
		HR (95 %CI)	*P* value	HR (95 %CI)	*P* value	HR (95 %CI)	*P* value
Day 1	40	1.08(1.01–1.17)	0.034	1.09 (1.01–1.17)	0.032	1.12 (1.02–1.23)	0.015
Day 2	40	1.04 (0.95–1.14)	0.365	1.04 (0.95–1.14)	0.419	1.09 (0.96–1.23)	0.188
Day 3	40	1.00(0.91–1.1)	0.989	1.00(0.91–1.11)	0.952	1.03 (0.91–1.17)	0.612
Day 4	40	1.04 (0.96–1.14)	0.318	1.05 (0.96–1.14)	0.267	1.06 (0.95–1.18)	0.305
Day 5	40	1.01 (0.93–1.1)	0.791	1.02 (0.94–1.11)	0.659	0.99 (0.89–1.1)	0.798

Model 1: unadjusted.

Model 2: adjusted for age and sex.

Model 3: adjusted for age, sex, white blood cell (WBC) count, respiratory rate, hypertension, diabetes, serum amyloid A (SAA), and soluble suppression of tumorigenicity 2 (sST2).

Abbreviations: HR, hazard ratio; CI, confidence interval.

### 3.5 Kaplan–Meier survival analysis

**Fig. 2:**
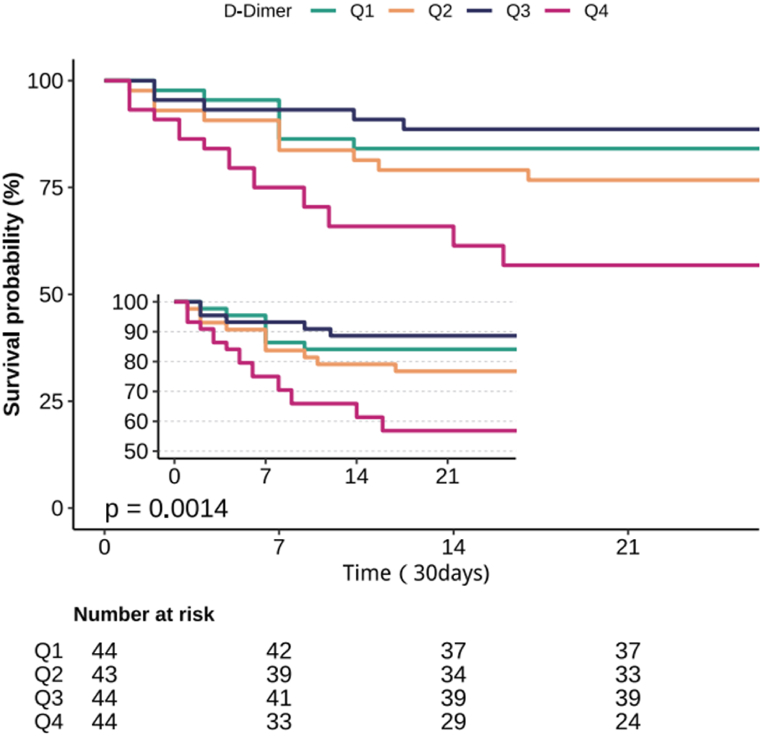
Kaplan-Meier survival curves for 30-day all-cause mortality in sepsis patients stratified by plasma D-dimer quartiles.

Kaplan–Meier survival analysis demonstrated that higher plasma D-dimer levels were significantly associated with reduced cumulative 30-day survival (Fig. 2; *P* = 0.0014). Patients in the highest D-dimer quartile (Q4) had the lowest survival probability compared to those in the lower quartiles.

Kaplan-Meier survival curves illustrating the 30-day all-cause mortality among sepsis patients stratified by plasma D-dimer quartiles (Q1 to Q4). The number of patients at risk in each quartile group is shown at specific time points. The log-rank test yielded a *P*-value of 0.0014, indicating a statistically significant difference in survival across the groups. Notably, patients in the highest D-dimer quartile (Q4) exhibited the lowest survival probability.

Q1–Q4 represent D-dimer quartile stratifications: Q1 (<1.26 μg/mL), Q2 (1.26–2.18 μg/mL), Q3 (2.18–5.38 μg/mL), and Q4 (>5.38 μg/mL). P values are from log-rank tests.

### 3.6 Subgroup analysis

**Fig. 3:**
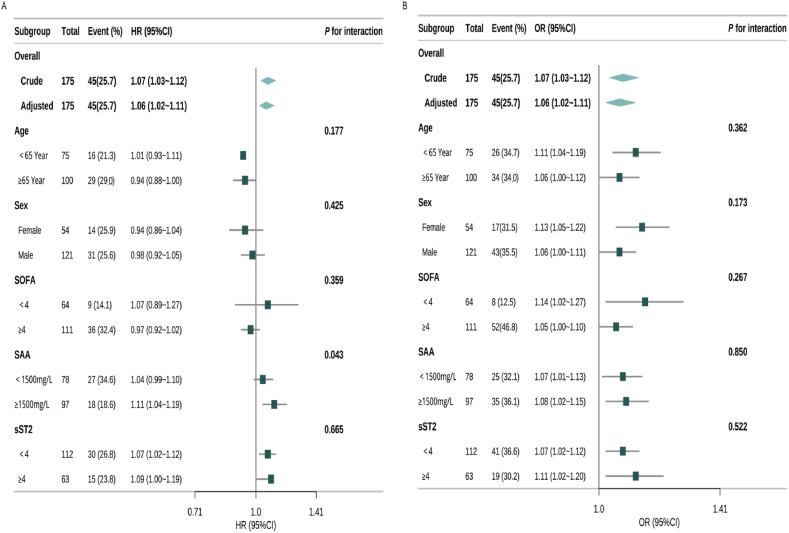
Subgroup analyses of the association between plasma D-dimer levels and clinical outcomes. SAA: serum amyloid A; sST2: soluble suppression of tumorigenicity 2; SOFA: Sequential Organ Failure Assessment; HR: hazard ratio; OR: odds ratio; CI: confidence interval; WBC: white blood cell. Forest plots displaying the adjusted HRs and ORs with 95 % confidence intervals for clinical outcomes across subgroups. Panel (A) shows the association between plasma D-dimer levels and 30-day all-cause mortality based on multivariable Cox regression models. Panel (B) presents the association between D-dimer levels and the risk of septic shock using logistic regression analysis. Subgroup analyses were stratified by age, sex, SOFA score, serum SAA, and sST2 levels. All models were adjusted for age, sex, WBC count, respiratory rate, hypertension, diabetes, serum SAA, and sST2. A significant interaction was observed in the SAA subgroup for 30-day mortality (*P* for interaction = 0.043), indicating potential effect modification. No significant interactions were detected for septic shock across any subgroups (all *P* for interaction >0.05). Diamonds represent overall effect estimates, and horizontal lines represent 95 % CIs.

Subgroup analysis of the association between plasma D-dimer levels and 30-day all-cause mortality (Fig. 3A**)** revealed a statistically significant interaction with serum SAA levels (interaction *P* = 0.043). However, no significant interactions were observed in subgroups defined by age, sex, SOFA score, or sST2 levels (all interaction *P* > 0.05).

In contrast, for the outcome of septic shock (Fig. 3B), the association between D-dimer levels and risk remained consistent across all predefined subgroups. No significant interactions were detected in analyses stratified by age, sex, SOFA score, SAA, or sST2 levels (all interaction *P* > 0.05). To explore the potential interaction between coagulation and inflammation in predicting 30-day mortality, we further conducted joint effect analysis of D-dimer and serum amyloid A (SAA) levels. As shown in Supplementary Fig. 2 and Supplementary Table 3, no statistically significant multiplicative or additive interaction was observed (P > 0.05).

## 4 Discussion

This study systematically evaluated the prognostic value of plasma D-dimer levels in critically ill patients with sepsis. Elevated D-dimer levels were independently associated with an increased risk of 30-day all-cause mortality and the incidence of septic shock. Subgroup analysis further revealed a potential interaction between serum amyloid A (SAA) levels and mortality risk, suggesting that concurrent evaluation of coagulation and inflammatory markers—such as D-dimer and SAA—may enhance prognostic assessment. These findings support the potential clinical utility of D-dimer as an accessible biomarker for early risk stratification in sepsis, though further validation is needed.

While D-dimer is widely recognized as a marker of coagulation activation, its prognostic value in sepsis remains under debate [10,16]. Previous studies have reported inconsistent findings regarding its association with adverse outcomes in septic patients [17,18]. Some identified elevated D-dimer as an independent predictor of mortality or disease severity, whereas others reported weak or non-significant associations.

For example, Schupp et al. (2023) found that D-dimer, when used alongside disseminated intravascular coagulation (DIC) scores, was useful in stratifying sepsis severity but had limited value for predicting mortality [10]. Other studies suggest that composite indices, such as the D-dimer-to-albumin ratio, may outperform D-dimer alone in prognostication [19,20]. Conversely, some research has emphasized the nonspecific nature of D-dimer elevation, noting that it may occur in a wide range of inflammatory or thrombotic conditions, thereby limiting its specificity [21,22]. Variations in study design, sample size, assay standardization, and statistical adjustment likely contribute to these discrepancies. Few studies have explored the interaction between coagulation and inflammatory biomarkers in sepsis prognosis.

To address these gaps, our study employed a prospective cohort design, included multivariable adjustments for key confounders, and incorporated subgroup analyses. This approach strengthens the observed association between D-dimer and both mortality and septic shock and suggests that D-dimer may serve as a standalone biomarker to support clinical risk stratification in sepsis. Although elevated D-dimer levels are not specific to sepsis and may reflect the overall severity of illness, they remain clinically relevant. D-dimer is a well-established marker of coagulation activation and systemic inflammation, and its elevation can occur in a wide range of conditions, including trauma, malignancy, and cardiovascular events. However, in our cohort, D-dimer levels measured at ICU admission were independently associated with both 30-day mortality and septic shock, even after adjusting for SOFA score, comorbidities, and other inflammatory markers (e.g., SAA, sST2). This suggests that despite its non-specificity, D-dimer provides valuable prognostic information beyond general illness severity in septic patients [23,24].

The observed relationship between elevated D-dimer levels and poor outcomes in sepsis is consistent with current understanding of sepsis pathophysiology. Sepsis is characterized by a dysregulated host response involving systemic inflammation and activation of the coagulation cascade [[25], [26], [27]]. Excessive thrombin generation, impaired fibrinolysis, and endothelial dysfunction may result in microvascular thrombosis, tissue hypoxia, organ dysfunction, and progression to septic shock [28,29]. Moreover, there is a well-established bidirectional interplay between inflammation and coagulation: inflammatory cytokines such as interleukin-6 (IL-6) and tumor necrosis factor-alpha (TNF-α) promote coagulation, while coagulation products further sustain inflammation [30]. These mechanisms may help explain why concurrent elevations in inflammatory and coagulation markers are associated with a higher risk of adverse outcomes. The results of this study suggest that combining such markers could improve early prognostic assessment in sepsis. Although the interaction between SAA and D-dimer was not statistically significant, stratified analysis revealed a trend-level difference, suggesting that their combined prognostic relevance warrants further investigation in larger cohorts.

The non-linear trend revealed by the RCS analysis helps to explain the decreased mortality risk observed in Q3. Moderately elevated D-dimer levels may represent an early or compensatory response to inflammation and coagulation imbalance, while markedly elevated levels are more likely to reflect persistent coagulopathy and worse prognosis. Thus, modeling D-dimer as a continuous and non-linear variable appears more appropriate than relying solely on categorical stratification. Additionally, the RCS curve exhibited a slight downward trend at extremely high D-dimer levels; however, this section involved limited sample size and wide confidence intervals, suggesting that the observed dip may result from statistical fluctuation rather than a true protective effect. This finding does not undermine the overall positive association between D-dimer levels and mortality.

This study has several strengths. It is among the few prospective cohort studies to evaluate the association between D-dimer and both mortality and septic shock in ICU patients with sepsis. The inclusion of inflammatory markers offers additional insight into the interplay between coagulation and immune responses. Moreover, D-dimer testing is widely available, cost-effective, and routinely used in clinical practice, enhancing the translational relevance of our findings for risk stratification and early intervention.

Nevertheless, several limitations should be acknowledged. First, this was a single-center study with a relatively small sample size, which may limit generalizability. However, the prospective design and standardized data collection enhance internal validity. Second, we assessed only baseline D-dimer levels and did not account for dynamic changes over time. Third, residual confounding cannot be fully excluded despite adjustment for multiple covariates. To address these limitations, we conducted subgroup and sensitivity analyses to assess the robustness of our results. Future multicenter studies with larger cohorts and longitudinal biomarker monitoring are warranted to validate and extend these findings.

## 5 Conclusion

In this prospective cohort study, elevated plasma D-dimer levels were independently associated with increased risks of 30-day all-cause mortality and septic shock in critically ill patients with sepsis. These findings indicate that D-dimer may serve as a practical and accessible biomarker for early risk stratification and prognostic assessment. Incorporating D-dimer levels at ICU admission into routine clinical assessment may facilitate the timely identification of high-risk patients and support individualized treatment decisions.

## CRediT authorship contribution statement

**Xiaoxiao Qu:** Writing – original draft, Data curation, Conceptualization. **Shishi Wang:** Formal analysis, Data curation. **Xuanmei Ye:** Investigation, Formal analysis. **Guosong Jiang:** Investigation, Data curation. **Mihereguli Kuerban:** Data curation. **Qipeng Xie:** Writing – review & editing, Conceptualization.

## Informed consent

Written informed consent was waived due to the use of anonymized, residual serum samples that were originally collected for routine clinical testing and subsequently reanalyzed for research purposes. No additional sampling or intervention was conducted, and all patient data were de-identified prior to analysis. The study was conducted in accordance with the Declaration of Helsinki and relevant institutional ethical guidelines.

## Ethical approval

This study was approved by the Ethics Committee of The Second Affiliated Hospital of Wenzhou Medical University (Approval No. 2023-K-234-01).

## Clinical trial number

Not applicable.

## Funding

This work was supported by grants from 10.13039/501100007194Wenzhou Science and Technology Bureau (Y2023774).

## Declaration of competing interest

The authors declare that they have no competing interests.

## Data Availability

Data will be made available on request.
