# Supplementary material for: Prognostic value of baseline plasma D-dimer levels in sepsis: a prospective cohort study
Source: Pract Lab Med. 2025 Aug 23;46:e00498. doi: 10.1016/j.plabm.2025.e00498 (PMC12398842; doi:10.1016/j.plabm.2025.e00498)
Supplement: Multimedia component 1 [file mmc1.docx]

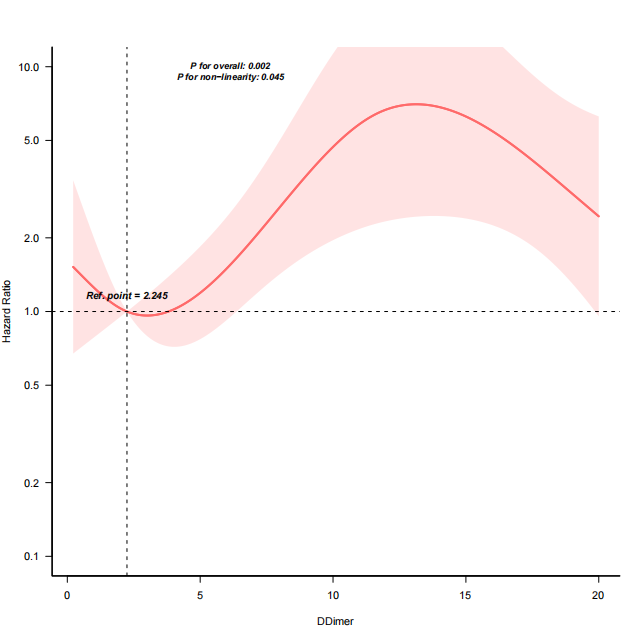


**Supplementary Figure 1.** Adjusted association between plasma D-dimer levels and 30-day mortality using restricted cubic spline (RCS) modeling.
The red line represents the estimated hazard ratio, and the shaded area indicates the 95% confidence interval. The reference point (HR = 1) was set at 2.245 µg/mL. The model was adjusted for age, sex, WBC count, respiratory rate, hypertension, diabetes, SAA, and sST2. The non-linear association was statistically significant (P for non-linearity = 0.045).


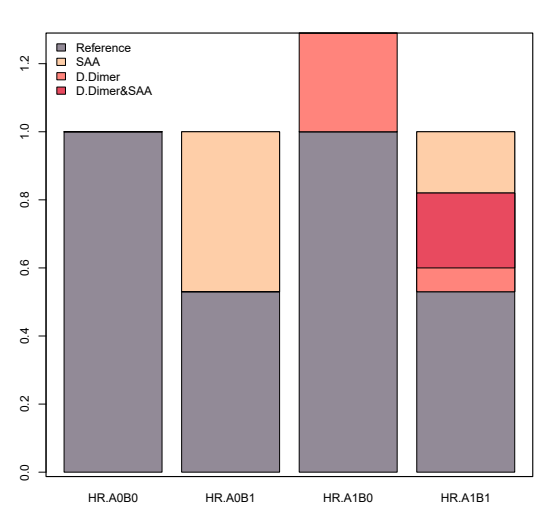

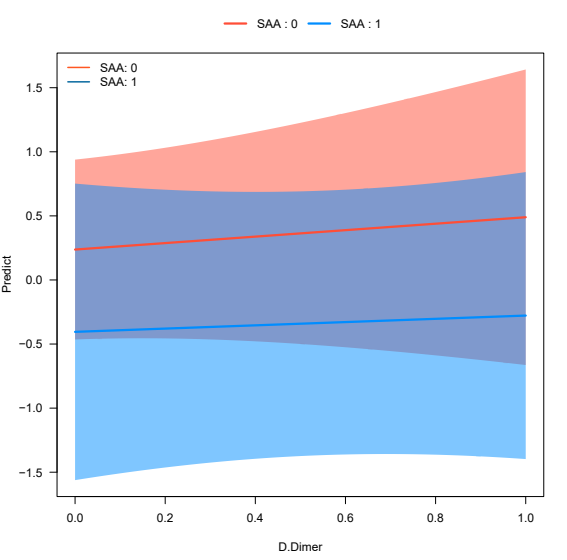
 **Supplementary Figure 2.** Interaction analysis between D-dimer and SAA for 30-day mortality.
**Panel A** shows relative hazard components for combinations of high/low SAA and D-dimer levels.**Panel B** shows predictive margins for D-dimer stratified by SAA status

**A B**
