# Supplementary material for: Prognostic value of baseline plasma D-dimer levels in sepsis: a prospective cohort study
Source: Pract Lab Med. 2025 Aug 23;46:e00498. doi: 10.1016/j.plabm.2025.e00498 (PMC12398842; doi:10.1016/j.plabm.2025.e00498)
Supplement: Multimedia component 2 [file mmc2.docx]

**Supplementary Table 1**. Association Between Daily Plasma D-dimer Levels (Day 1–5) and the Risk of Septic Shock in ICU Patients With Sepsis

Model 1: unadjusted;

Model 2: adjusted for age and sex;

Model 3: adjusted for age, sex, white blood cell (WBC) count, respiratory rate, hypertension, diabetes, serum amyloid A (SAA), and soluble suppression of tumorigenicity 2 (sST2).

Abbreviations: OR, Odds Ratio; CI, confidence interval.

| D-dimer Day | N | Model 1 | | Model 2 | | Model 3 | |
| --- | --- | --- | --- | --- | --- | --- | --- |
|  |  | OR (95%CI) | *P* value | OR (95%CI) | *P* value | OR(95%CI) | *P* value |
| Day 1 | 40 | 1.00  (0.91~1.1) | 0.98 | 0.97 (0.87~1.08) | 0.553 | 0.96 (0.86~1.07) | 0.450 |
| Day 2 | 40 | 1.09  (0.97~1.21) | 0.136 | 1.08 (0.96~1.21) | 0.195 | 1.06  (0.93~1.2) | 0.369 |
| Day 3 | 40 | 1.07  (0.96~1.19) | 0.206 | 1.05 (0.93~1.17) | 0.43 | 1.03 (0.91~1.16) | 0.678 |
| Day 4 | 40 | 1.05  (0.95~1.16) | 0.333 | 1.04 (0.94~1.15) | 0.267 | 1.02 (0.91~1.14) | 0.760 |
| Day 5 | 40 | 1.05  (0.96~1.14) | 0.326 | 1.04 (0.94~1.14) | 0.455 | 1.02 (0.91~1.14) | 0.719 |

Supplementary Table 2. Sensitivity analysis of the association between plasma D-dimer and 30-day mortality after additional adjustment for serum creatinine

| Categories | Model 1 | | Model 2 | | Model 3 | |
| --- | --- | --- | --- | --- | --- | --- |
|  | HR (95%CI) | *P* value | HR (95%CI) | *P* value | HR (95%CI) | *P* value |
| D-Dimer | 1.07 (1.03~1.12) | 0.001 | 1.07 (1.03~1.12) | 0.001 | 1.06 (1.02~1.11) | 0.007 |
| D-Dimer Quartile |  |  |  |  |  |  |
| Q1 (<1.26) | 1(Ref) |  | 1(Ref) |  | 1(Ref) |  |
| Q2 (1.26~2.18) | 1.31 (0.54~3.16) | 0.547 | 1.31 (0.54~3.17) | 0.544 | 1.1 (0.44~2.75) | 0.845 |
| Q3 (2.18~5.38) | 0.54 (0.18~1.62) | 0.272 | 0.51 (0.17~1.52) | 0.225 | 0.36 (0.11~1.16) | 0.088 |
| Q4 (>5.38) | 2.69 (1.23~5.92) | 0.014 | 2.64 (1.19~5.83) | 0.017 | 2.34 (1.02~5.36) | 0.044 |

Model 1: unadjusted;

Model 2: adjusted for age and sex;

Model 3: adjusted for age, sex, WBC count, respiratory rate, hypertension, diabetes, serum amyloid A (SAA), and soluble suppression of tumorigenicity 2 (sST2),creatinine.

Abbreviations: HR, hazard ratio; CI, confidence interval; WBC, white blood cell count.

**Supplementary Table 3.** Interaction analysis between D-dimer and SAA in predicting 30-day mortality, using both multiplicative and additive models.

| Measures | Estimates | CI.95.low | CI.95.UP | P.value |
| --- | --- | --- | --- | --- |
| HR00 | 1 | － | － | － |
| HR01 | 0.53 | 0.21 | 1.35 | 0.18 |
| HR10 | 1.29 | 0.57 | 2.91 | 0.54 |
| HR11 | 0.6 | 0.26 | 1.38 | 0.23 |
| HR(SAA on outcome [D.Dimer==0] | 0.53 | 0.21 | 1.35 | 0.18 |
| HR(SAA on outcome [D.Dimer==1] | 0.46 | 0.2 | 1.07 | 0.07 |
| HR(D.Dimer on outcome [SAA==0] | 1.29 | 0.57 | 2.91 | 0.54 |
| HR(D.Dimer on outcome [SAA==1] | 1.14 | 0.44 | 2.96 | 0.81 |
| Multiplicative scale | 0.88 | 0.25 | 3.07 | 0.84 |
| RERI | -0.22 | -1.36 | 0.93 | 0.64 |
| AP | -0.36 | -2.24 | 1.52 | 0.35 |

Note: Certain figures and tables related to subgroup and interaction analyses (e.g., between D-dimer and SAA) are provided within the response letter for reviewer reference only and are not included in the revised manuscript or supplementary files.
